# Toward Light-Controlled Supramolecular Peptide Dimerization

**DOI:** 10.1021/acs.joc.1c00464

**Published:** 2021-06-01

**Authors:** Rita J. Fernandes, Patricia Remón, Artur J. Moro, André Seco, Ana S. D. Ferreira, Uwe Pischel, Nuno Basílio

**Affiliations:** † Laboratorio Associado para a Química Verde (LAQV), Rede de Química e Tecnologia (REQUIMTE), Departamento de Química, Faculdade de Ciências e Tecnología, Universidade Nova de Lisboa, 2829-516 Caparica, Portugal; ‡ CIQSO - Centre for Research in Sustainable Chemistry and Department of Chemistry, University of Huelva, Campus de El Carmen s/n, E-21071 Huelva, Spain; § UCIBIO, REQUIMTE, Departamento de Química, Faculdade de Ciências e Tecnologia, Universidade Nova de Lisboa, 2829-516 Caparica, Portugal

## Abstract

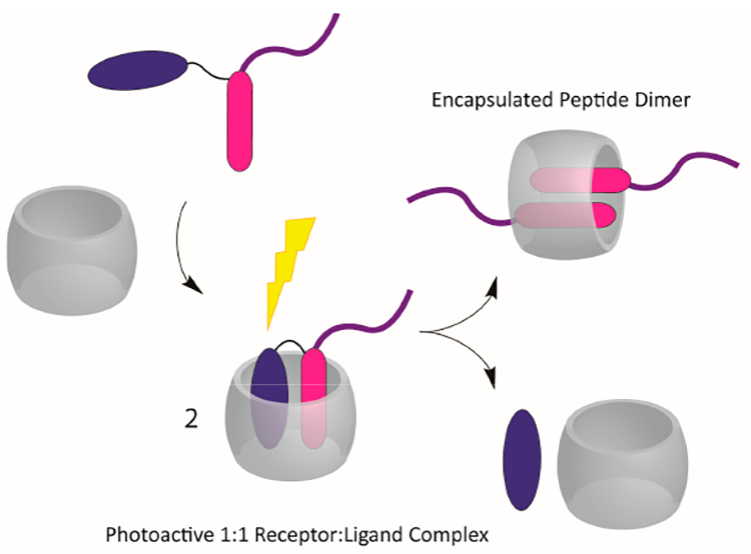

The selective photodeprotection of the NVoc-modified FGG tripeptide yields the transformation of its 1:1 receptor–ligand complex with cucurbit[8]uril into a homoternary FGG_2_@CB8 assembly. The resulting light-induced dimerization of the model peptide provides a tool for the implementation of stimuli-responsive supramolecular chemistry in biologically relevant contexts.

It is widely recognized that the biological function of most proteins in a cellular environment is governed by the formation of self-assembled complexes with other proteins and that the dysregulation of these processes may lead to pathological conditions.^1,2^ Synthetic small molecular receptors/ligands, capable of targeting protein hotspots/domains with high affinity and selectivity, are thus of key importance due to their potential to act as inhibitors or modulators of protein–protein interactions.^1−5^ Moreover, these small molecules can constitute valuable tools to trigger aggregation/disaggregation processes and to investigate the corresponding response.^6,7^

Among the different synthetic receptors that can be employed to recognize amino acid residues in peptides and proteins, cucurbit[*n*]urils (CB*n*) are particularly interesting due to their unmatched binding properties toward size- and shape-complementary organic molecules in aqueous solution.^8−15^ The high binding affinity and selective recognition of specific peptide sequences is particularly impressive and has revealed promising potential in applications such as drug delivery, enzymatic assays, supramolecular functionalization of proteins, sequence-specific protease inhibition, sensing, and protein purification.^16−33^ Of particular relevance is the use of cucurbit[8]uril (CB8) for the high-affinity inclusion of two phenylalanine residues in its cavity. This has been exploited as a powerful supramolecular tool to promote the dimerization and higher-order aggregation of proteins and peptides.^17,26,28,34−39^

The use of light to control functions in supramolecular systems, such as stimuli-responsive cucurbituril receptor–ligand complexes,^40−48^ or in biological contexts^49,50^ is highly appealing because of the unparalleled spatiotemporal resolution and the possibility of remote triggering. Likewise, the modulation of biological functions by light has received wide attention as a potential means to overcome shortcomings such as undesired endogenous reactions and off-target effects.^51−54^

Inspired by these modern developments at the intersection of supramolecular and biological chemistry, we became interested in exploring a way to trigger supramolecular peptide dimerization by means of light as an external stimulus. Given the popularity of the phenylalanine–glycine–glycine (FGG) motif in CB8-induced protein dimerization,^34−38^ we decided to use this tripeptide as the model system in our study. The concentration- and sequence-dependent switching between 1:1 and 2:1 ligand–CB8 complexes with short peptides has been investigated before but never in the context of light-triggered processes.^17,21,28^ Recently, we reported that CB8-templated FGG dimers can be dissociated by means of a light-induced competitive displacement.^45^ The demonstration of the *inverse* process, the light-induced formation of supramolecular FGG dimers, has remained elusive so far. In this work, we adopted an approach consisting of the NVoc-modified FGG (NVoc-FGG; NVoc is 6-nitroveratryloxycarbonyl; see Experimental Section for details on the synthesis and the Supporting Information for ^1^H and ^13^C NMR spectra, Figures S1–S5, as well as the high-resolution mass spectrum, Figure S6), which can be deprotected upon light irradiation to form the FGG tripeptide (see Scheme 1). NVoc-FGG binds to CB8 with a 1:1 stoichiometry. However, upon photodeprotection, this complex is converted into the stable homoternary FGG_2_@CB8 complex, thus providing a proof-of-principle for a simple and versatile strategy to trigger the CB8-assisted dimerization of peptides with light (Scheme 1). In addition, it is a frequently identified problem that the dimerization trigger (i.e., the receptor macrocycle in our case) has to be made available at the recognition site, involving eventual complications due to transport and diffusion in complex environments.^55,56^ Importantly, in our approach, the strong association of NVoc-FGG with CB8 assures the spatial colocalization of the macrocycle and the FGG prior to light stimulation.

**Scheme 1:**
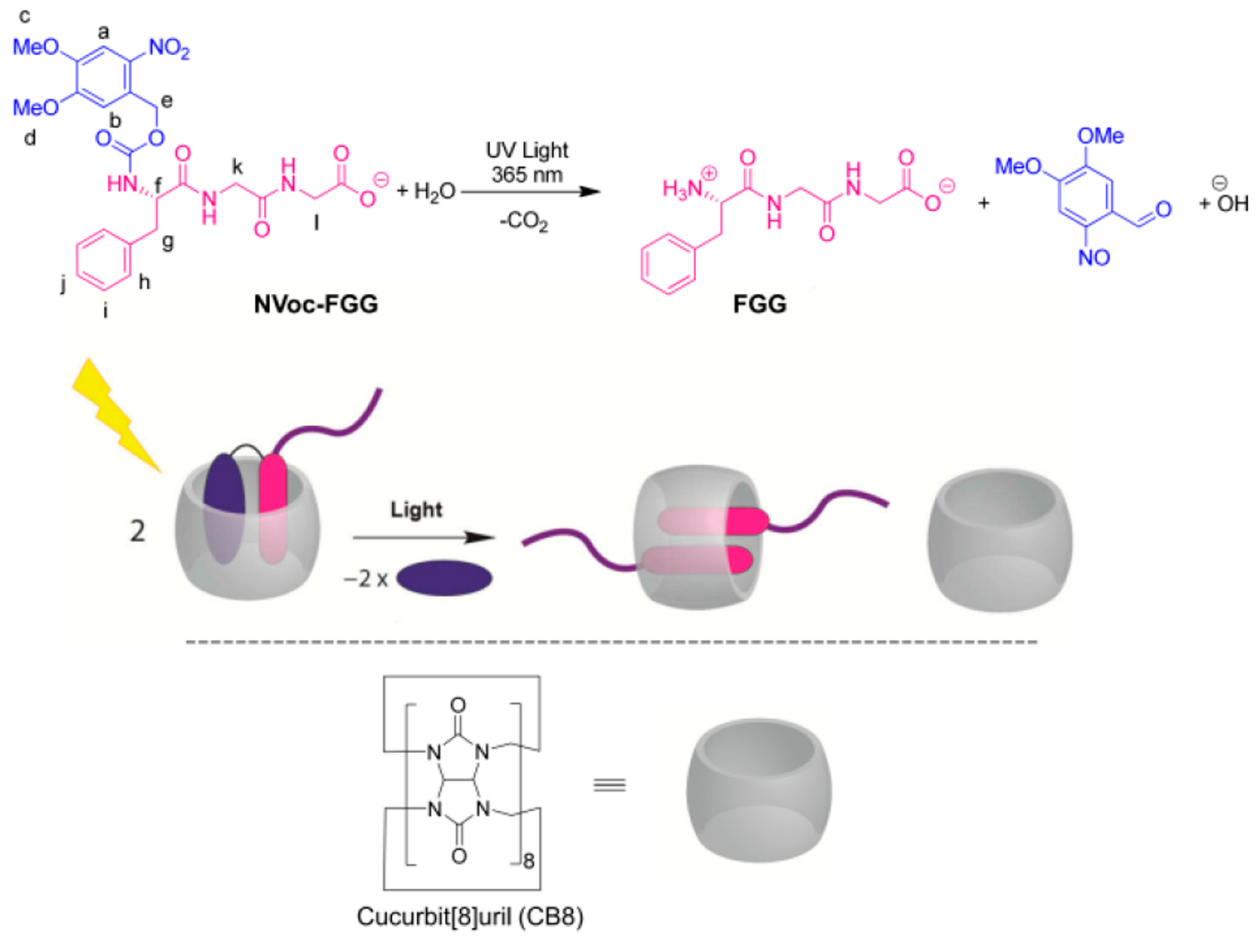
Photoinduced Supramolecular Dimerization of FGG Peptide Mediated by the Light-Triggered Transformation of a 1:1 into a 2:1 Ligand–ReceptorComplex, Formed between CB8 and NVoc-FGG or FGG Note that NVoc-FGG is deprotonated and FGG is present in its zwitterionic form at the neutral pH employed in this work.

Direct proof for the binding of NVoc-FGG by CB8 was obtained from isothermal titration experiments (ITC); see Figure 1. The ITC data are compatible with 1:1 binding as seen from the inflection of the titration curve at ∼1 equiv of titrant. The obtained 1:1 binding constant (at 25 °C) of *K*_11_ = 1.8 × 10^6^ M^–1^ in neutral water is in very satisfactory agreement with UV/vis absorption titrations (see Figures S7 and S8 in the Supporting Information). The ITC experiment was repeated in 10 mM sodium phosphate buffer (pH 7.4) at 25 °C (see Figure S9 in the Supporting Information), which returned a binding constant of 6.1 × 10^5^ M^–1^. This value is somewhat smaller than that for unbuffered aqueous solution (see above), which is reasoned to be due to the presence of competitively binding sodium ions in the buffer. In stark contrast to the 1:1 binding of NVoc-FGG, the ITC data for the titration of CB8 with FGG clearly show a profile compatible with a sequential 2:1 binding scenario.^17,57^ Considering the effects of competitive sodium cation binding,^58^ the obtained overall ternary binding constant of *K*_11_*K*_12_ = 1.7 × 10^12^ M^–2^ in unbuffered water (at 25 °C) is in good agreement with literature data for 10 mM sodium phosphate buffer (*K*_11_*K*_12_ = 1.5 × 10^11^ M^–2^ at pH 7.0, 27 °C).^17^ The stepwise binding constants show a high uncertainty, and for the same reasons as stated earlier by Urbach and co-workers, we report here just the overall 2:1 binding affinity.^17^ The binding of NVoc-FGG is clearly enthalpy-driven (Δ*H* = −41.5 kJ mol^–1^, −*T*Δ*S* = 7.3 kJ mol^–1^; in neutral water at 25 °C), being indicative of good shape and size complementarity between the receptor cavity and the included ligand moiety.^21,28,59,60^ In the case of FGG, reliable Δ*H* values for the separate 1:1 and 2:1 binding events could not be obtained due to the observed strong correlation between these two parameters. The stoichiometries of the NVoc-FGG@CB8 and FGG_2_@CB8 complexes were also detected by mass spectrometry (see Figures S10 and S11 in the Supporting Information).

**Figure 1:**
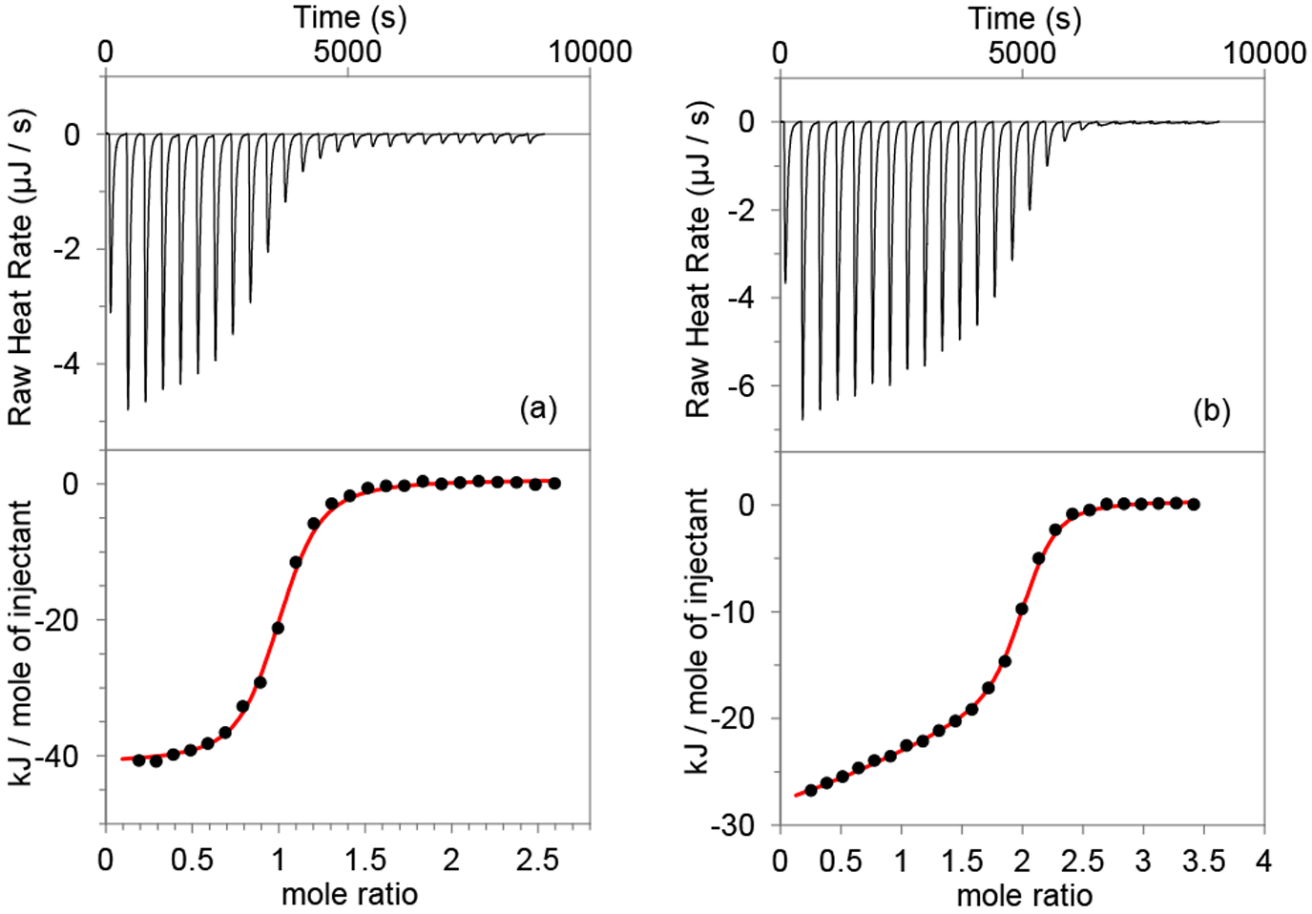
Isothermal titration calorimetry data for (a) titration of 80 μM CB8 with 1.5 mM of NVoc-FGG in neutral water and for (b) 120 μM CB8 titrated with 3.0 mM of FGG in neutral water.

CB*n* macrocycles are widely recognized as ideal receptors for organic cations. However, it has been demonstrated that the enthalpic nonclassical hydrophobic effect, i.e., the energetic gain associated with the release of high-energy water molecules from the receptor cavity upon ligand binding, is the major driving force responsible for the often high affinity for complementary ligands.^61^ Thus, ligands containing hydrophobic segments that match the CB*n* cavity in terms of size and shape usually display significant binding constants.^59,62^ Reasoning with the well-known ability of CB8 to include two ligands in its cavity,^9^ the simultaneous efficient inclusion of the adjacent hydrophobic phenyl and NVoc moiety is highly reasonable (see also NMR studies below). Similar observations were reported previously for the concomitant CB8 binding of neighboring amino acids of oligopeptides.^18,21,28,63^

At this point, it is constructive to contrast the CB8 binding of NVoc-FGG with the behavior of NVoc-Phe (NVoc-phenylalanine). The latter does not bind to CB8 with a significant affinity; no heat release was seen in the ITC experiment, and also no significant changes were observed in the UV/vis absorption spectrum of NVoc-Phe in the presence of CB8 (see Figures S12 and S13 in the Supporting Information). Noteworthy, Phe binds with a homoternary binding constant 4 orders of magnitude lower than that of FGG (*K*_11_*K*_12_ = 5.0 × 10^8^ M^–2^ for Phe; see Figure S13 in the Supporting Information). This underpins the stabilizing role of the glycine rests in FGG, as visualized in a previously reported crystal structure,^17^ showing dipole–dipole interactions of the amide NHs with receptor portal carbonyl groups.

Compelling evidence for the proposed 1:1 binding mode of NVoc-FGG was obtained from ^1^H NMR titration experiments. Upon addition of CB8 to the NVoc-FGG ligand, new ^1^H NMR signals, assigned to the complex, appear in slow exchange with those of the free ligand. The titration data and the complete assignment of the signals can be found in the Supporting Information (Figures S15–S17). Reaching 1 equiv of CB8, the signals of the free ligand completely disappear and no further changes are observed upon addition of excess CB8. This is in accordance with the formation of a stable 1:1 complex. Noteworthy, the assembly also shows several signals under chemical exchange, which were assigned to two rotamers arising from the slow rotation around the N–C carbamate (N–C=O) bond (Figure S14); see Supporting Information for a detailed discussion. The ^1^H NMR signals of the phenylalanine part and NVoc groups appear significantly shifted to higher field, in agreement with the concomitant inclusion of the two moieties in the receptor cavity. The proton signals of the NVoc methoxy groups are shifted to lower field, indicating their localization near the CB8 carbonyl portals. The ^1^H NMR titration of FGG with CB8 (see Figure S18 in the Supporting Information) is in line with the previously established formation of a homoternary complex.^17^

The formation of higher-order 2:2 (or *n*/*n*) receptor–ligand complexes with CB8 has been recently identified as a potential pitfall in the analysis of such systems, which may lead to the erroneous assignment of a 1:1 binding stoichiometry.^64^ In order to discard this possibility in the case of the NVoc-FGG@CB8 complex, we rely on a DOSY experiment (see Figures S19 and S20 in the Supporting Information). This afforded a diffusion coefficient of *D*_obs_ = 2.9 × 10^–10^ m^2^ s^–1^, which is in agreement with a 1:1 binding stoichiometry. For 2:2 complexes, *D*_obs_ would be expected to be below 2.1 × 10^–10^ m^2^ s^–1^.^28^

Having the transformation from 1:1 to 2:1 binding stoichiometry on changing from NVoc-FGG to FGG unambiguously established, we proceeded to demonstrate the light-induced supramolecular dimerization of FGG. The light irradiation (366 nm) of a solution of NVoc-FGG in the absence or presence of CB8 (1.4 equiv) yields UV/vis spectral variations (observed for the NVoc-derived photoproduct buildup at 415 nm) that are compatible with the removal of the NVoc group from the FGG tripeptide (see Figures S21 and S22 in the Supporting Information). It can be safely assumed that the mechanism of the photoreaction follows the same path as that derived from detailed transient absorption studies of related NVoc derivatives.^65^ Noteworthy, 366 nm light irradiation has been proven to be applicable in photodeprotections in biological contexts.^66^ However, if required, *o*-nitrobenzyl photochemistry can also be conducted with less energetic NIR light implied in two-photon excitation processes.^67,68^ Monitoring the photochemical reaction by ^1^H NMR spectroscopy clearly evidenced the formation of FGG from NVoc-FGG; see Figure S23 in the Supporting Information. The absence of the NVoc photoproduct signals could be explained by microprecipitation, which, however, was not visible to the naked eye. The photoreaction quantum yield, Φ, was determined to be 0.2% for NVoc-FGG (water, neutral pH, 366 nm irradiation), which is in agreement with the findings for other systems that contain this photoremovable group.^66,69^ However, for the corresponding CB8 complex, the quantum yield is reduced by 1 order of magnitude (Φ = 0.02%). The reasons for this behavior are not entirely clear, but we infer that the intracomplex proximity of the NVoc chromophore with the aromatic moiety of the phenylalanine moiety could lead to excited-state quenching. A comparison of the photoreaction kinetics, monitored by UV/vis absorption spectroscopy, did not show a very significant dependence of the photoreactivity on the presence of sodium phosphate buffer; see Figures S21 and S22 in the Supporting Information.

Direct demonstration of the photoinduced dimerization of FGG (i.e., formation of the FGG_2_@CB8 complex) was provided by ^1^H NMR experiments. The irradiation of a solution containing the 1:1 NVoc-FGG@CB8 complex (Figure 2a) yields a new set of signals that is readily assigned to the homoternary FGG_2_@CB8 complex (Figure 2b) by comparison with the corresponding reference spectrum (Figure 2c). Further support for the light-triggered formation of the FGG_2_@CB8 complex was obtained by irradiating an NVoc-FGG solution, containing a limiting quantity of 0.5 equiv of CB8 (see Figure S24 in the Supporting Information). First, two sets of signals, corresponding to free and 1:1 complexed NVoc-FGG in slow exchange at the NMR time scale, were observed. Upon irradiation, the ^1^H NMR signals of free and complexed NVoc-FGG vanish, giving rise to exclusively FGG_2_@CB8 resonances.

**Figure 2:**
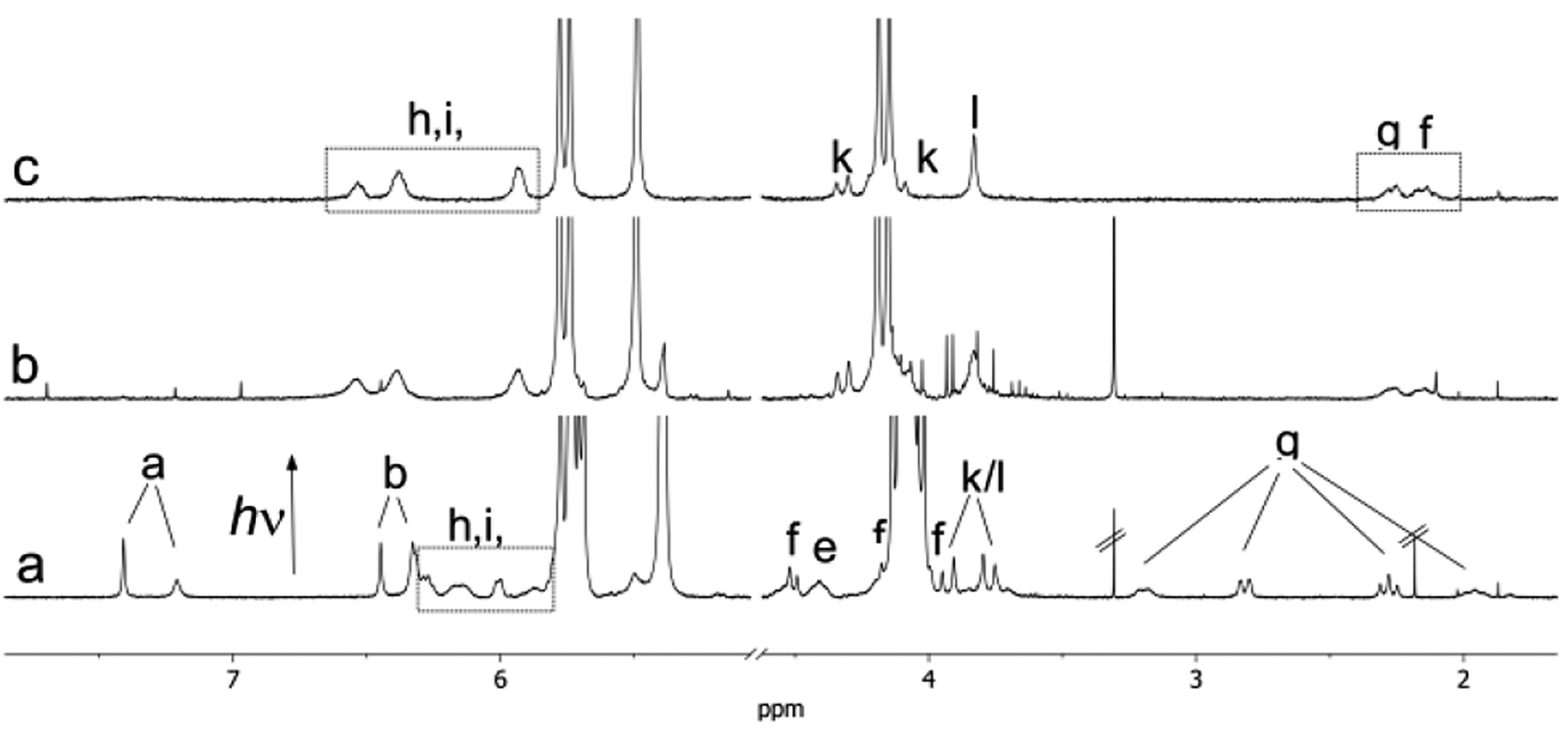
Partial ^1^H NMR spectra (400 MHz, D_2_O, 25 °C) of NVoc-FGG (200 μM) in the presence of 1 equiv of CB8 (200 μM) before (a) and after (b) photolysis at >300 nm (200 W Xe–Hg lamp); see Scheme 1 for assignment letters. Spectrum (c) corresponds to a solution of 500 μM FGG and 500 μM CB8 and is used for comparison. The assignment of the FGG_2_@CB8 complex was previously reported.^17^

In summary, we have delivered a proof-of-principle example for the photoinduced supramolecular dimerization of FGG peptide in aqueous solution, which consists of the protection of N-terminal phenylalanine with the photolabile NVoc group. This conjugate forms a strong 1:1 complex with CB8 that upon photodeprotection leads to a supramolecular peptide dimer encapsulated in CB8 (FGG_2_@CB8). The light-activated formation of this complex is of significant importance for protein recognition and constitutes a versatile tool for implementing stimuli-responsive supramolecular chemistry in biorelevant contexts. We anticipate that the general nature of the approach may increase the scope in terms of the peptide and photoprotection groups that could be employed.

## Experimental Section

### Materials

Phenylalanine–glycine–glycine (FGG) in its enantiomeric l-form, phenylalanine (Phe), and 6-nitroveratryloxycarbonyl chloride (NVoc) are commercially available and were used as received. The same applies to the solvents and reagents for synthesis. Cucurbit[8]uril (CB8), 3-amino-1-adamantanol (S1; see competitive displacement titration in the Supporting Information), and NVoc-Phe were available from previous studies.^42,44^

### Synthesis and Characterization of NVoc-FGG

The enantiomerically pure l-form of FGG peptide (0.192 g; 0.687 mmol) and sodium carbonate (0.083 g; 0.786 mmol) were dissolved in 8 mL of Milli-Q water. Then 4,5-dimethoxy-2-nitrobenzyl chloroformate (0.138 g; 0.500 mmol), dissolved in 8 mL of 1,4-dioxane, was added to the aqueous peptide solution, and the mixture was stirred for 2 h at 60 °C in an aluminum heating block. The reaction mixture was quenched by addition of 1 M HCl until reaching a pH of ∼2, and the crude product was extracted with dichloromethane (3 × 20 mL). After being dried with anhydrous Na_2_SO_4_, the organic phase was concentrated by rotary evaporation. The crude was purified by silica gel flash chromatography using an elution gradient of 100% chloroform to (v/v) 70% chloroform/30% MeOH. This procedure yielded the final product in 27% yield (yellow amorphous solid, 0.069 g): ^1^H NMR (400 MHz, CD_3_OD) δ (ppm) = 7.73 (s, 1H), 7.27–7.19 (m, 5H), 7.08 (s, 1H), 5.43 (dd, 2H), 4.39–4.35 (m, 1H), 3.95–3.68 (m, 10H), 3.18 (dd, 1H), 2.94 (dd, 1H); ^13^C{^1^H} NMR (101 MHz, CD_3_OD) δ (ppm) = 173.3, 171.5, 170.4, 156.8, 153.9, 148.1, 139.3, 137.1, 128.9 (2×), 128.1, 128.0, 126.4, 109.5, 107.8, 63.2, 56.9, 55.6, 55.4, 42.0, 40.3, 37.1; HRMS (ESI) *m*/*z* calcd for C_23_H_26_N_4_O_10_Na [M + Na^+^] 541.1541; found 541.1533.

### General Methods

The pH of the solutions, prepared with Milli-Q water, was adjusted with HCl or NaOH and measured with a Crison basic 20+ pH meter. The NMR experiments were run on a Bruker Avance III operating at 400 MHz (^1^H) or 100 MHz (^13^C). The solutions for the NMR experiments were prepared in D_2_O, and the pD was adjusted with DCl or NaOD. Corrections due to isotope effects were applied using the equation pD = pH* + 0.4, where pH* is the reading taken from the pH meter.^70^ Structural assignments were made with additional information from gCOSY and gROESY experiments. ^1^H COSY experiments were done using a gradient-selected COSY pulse sequence (cosygpqf), in which 16 transients were collected with a spectral window of ∼3400 Hz, 2 K data points in F2 and 128 data points in F1, and a relaxation delay of 2.0 s. The ^1^H–^1^H ROESY experiments implied a phase-sensitive pulse sequence (roesyphpp.2) with a spin-lock pulse of 300 ms. Thirty-two transients were collected with a spectral width of ∼2800 Hz; 2 K data points in F2 and 256 data points in F1. The relaxation delay was set to 2.0 s. Mass spectra (positive mode) were obtained with an Orbitrap Elite mass spectrometer (Thermo Scientific), equipped with a heated electrospray ionization source (HESI-II) and an FTMS orbitrap mass analyzer. UV/vis absorption spectra were recorded using a Varian Cary 100 Bio or a Shimadzu UV-1603 spectrophotometer.

### DOSY Experiments

^1^H NMR diffusion experiments (DOSY) were done with the stimulated echo sequence using bipolar sine gradient pulses (ledbpgp2s). For each experiment, the pulsed gradients were applied with a power level (*G*) linearly incremented from 2.65 to 50.4 G cm^–1^. The duration of the pulse field gradients (δ), applied to encode and decode the diffusion, was set to 4 ms, and the diffusion delay period (Δ) of the experiment was optimized to 150 ms. The optimized value of Δ provided a convenient sampling of the exponential decay of the signal intensity during the diffusion experiment. A shape factor of 0.6366 (ξ) was used to correct the gradient deviation arising from the use of sine-pulsed gradients. The integral of selected ^1^H NMR signals (*I*) was plotted against the gradient strength, and the data were fitted to the Stejskal–Tanner equation (see Supporting Information), where γ is the gyromagnetic ratio of the observed nucleus, to obtain the diffusion coefficient (*D*); see Figures S19 and S20.^71^

### Isothermal Titration Calorimetry

The experiments were performed on a Nano ITC (TA Instruments) with standard volumes. The solutions were thoroughly degassed before use by stirring under vacuum. The sample cell was loaded with the CB8 solution, and a 250 μL autopipette was filled with the ligand solution. The receptor was titrated in a sequence of either 50 injections of 5 μL or 25 injections of 10 μL after reaching baseline stability.

### Photochemistry

Light irradiation experiments were conducted with a 200 W Hg–Xe lamp, connected to a Newport power supply, using a 300 nm cutoff filter or a 366 nm band-pass filter. Our custom photochemical setup consists on the aforementioned lamp mounted on a Melles Griot optical table. The samples were irradiated at a 40 cm distance from the light source either in a 1 cm quartz cuvette or directly in a NMR tube, depending on if the progress of the reaction was being monitored by UV/vis absorption spectroscopy or by NMR spectroscopy, respectively.

HPLC analyses were carried out with a Merck Hitachi L6200A apparatus equipped with a DAD L-4500 Merck Hitachi detector. A Purospher Star RP18e 250 × 4 mm (5 μm) column was used, and the analyses were made using an acidified water (w/3% HClO_4_)/methanol gradient mixture ranging from 10 to 100% organic phase over a period of 30 min. For the quantum yield determinations, small aliquots of 30 μL were taken at given time intervals and injected in the chromatographic system using a 20 μL steel loop. An absorption wavelength of 220 nm was chosen for monitoring since it allowed the simultaneous detection of NVoc-FGG and FGG. A calibration curve for FGG was made by preparing a stock solution and injecting aliquots of different dilutions. This allowed for the quantification of FGG formed during the irradiations so that the quantum yield could be determined according to the following equation:
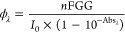
where Φ_λ_ is the photochemical quantum yield at the irradiation wavelength λ; *n*FGG is the amount (in moles) of formed product during the irradiation time (calculated from HPLC data); *I*_0_ photon flux reaching the sample, and Abs_λ_ is the absorbance at the irradiation wavelength λ.

## Supporting Information Available

NMR spectra of NVoc-FGG; additional CB8-binding data for NVoc-FGG, NVoc-Phe, and Phe; NMR and mass spectrometry characterization of the inclusion complexes; photochemical experiments monitored by UV/vis absorption spectroscopy and NMR spectroscopy (PDF)
