# Supplementary material for: Toward Light-Controlled Supramolecular Peptide Dimerization
Source: J Org Chem. 2021 Jun 1;86(12):8472–8. doi: 10.1021/acs.joc.1c00464 (PMC9161448; doi:10.1021/acs.joc.1c00464)
Supplement: Supplementary file 1 — jo1c00464_si_001.pdf [file jo1c00464_si_001.pdf]

Supporting Information

Towards Light-Controlled Supramolecular Peptide Dimerization

Rita J. Fernandes,[a] Patricia Remón,[b] Artur J. Moro,[a] André Seco,[a] Ana S. D. Ferreira,[c] Uwe Pischel,*[b] and Nuno Basílio*[a]

Table of Contents	S2
1. Characterization data for NVoc-FGG	S5
2. Additional experiments	S5
2.1. Direct UV/vis absorption titration	S5
2.2. Competitive displacement titration	S6
2.3. Isothermal titration calorimetry of NVoc-FGG/CB8 in buffered solution	S7
2.4. Mass spectra of the inclusion complexes	S8
2.5. Phenylalanine (Phe)/NVoc-Phe interactions with CB8	S9
2.6. NMR characterization of the inclusion complexes	S10
2.7. DOSY experiments	S14
2.8. Photochemistry	S16
3. References	S18

1. Characterization data for NVoc-FGG

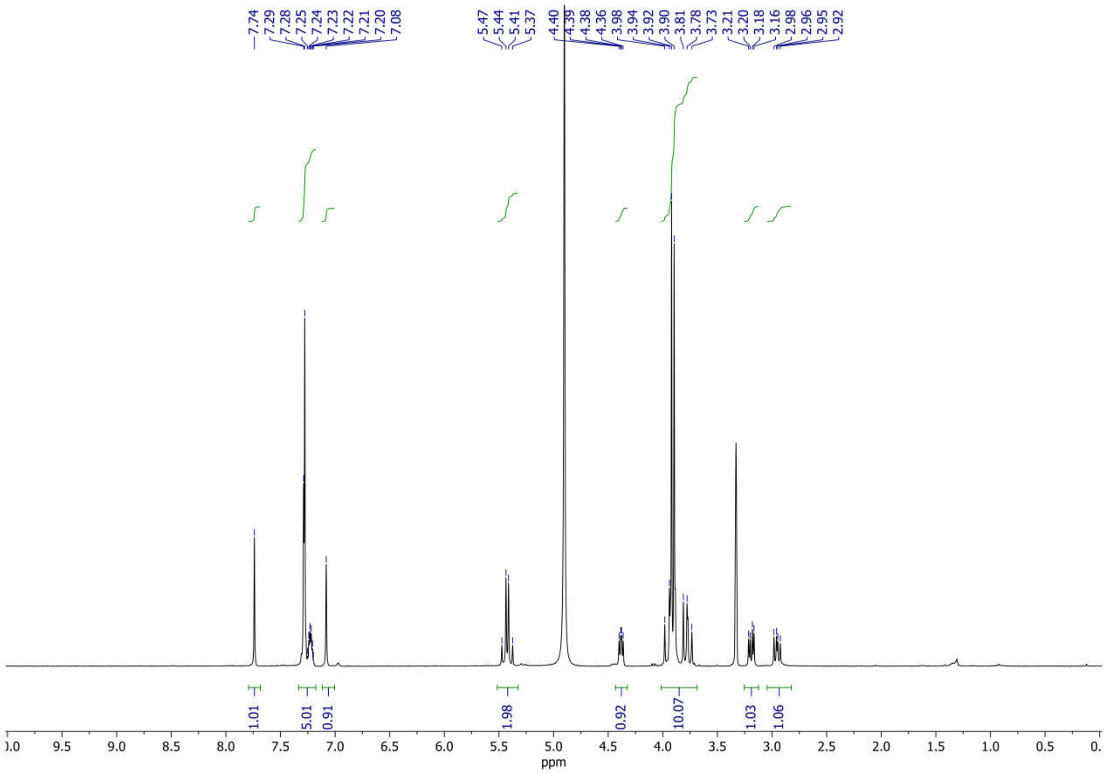

Figure S1. ^{1}H NMR (400 MHz) spectrum of **NVoc-FGG** in CD_3OD.

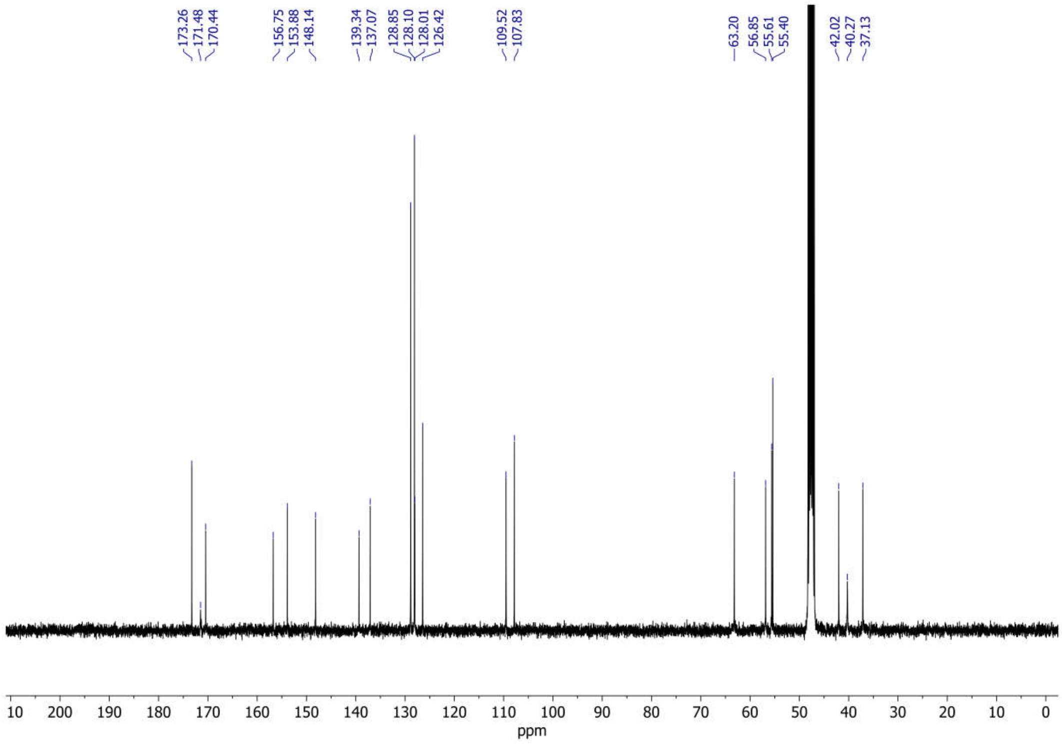

Figure S2. $^{13}C\{^{1}H\}$ NMR (101 MHz) spectrum of **NVoc-FGG** in CD_3OD.

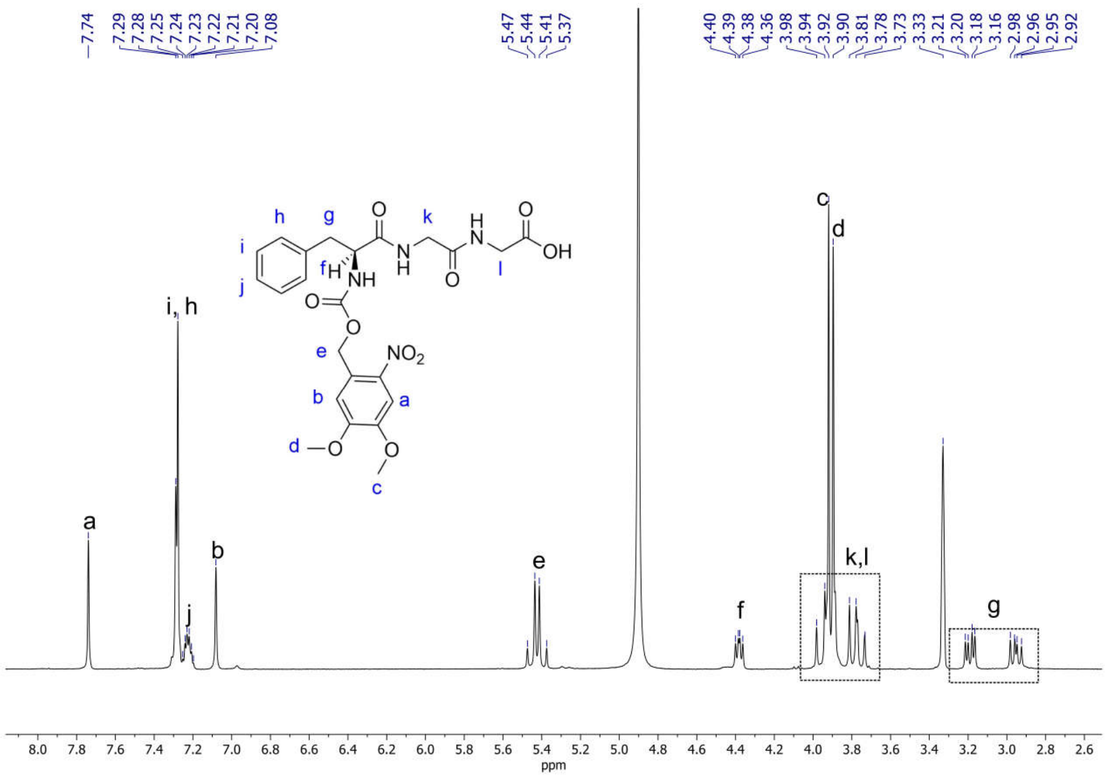

Figure S3. Partial ^{1}H NMR (400 MHz) spectrum of **NVoc-FGG** in CD_3OD showing the signal assignments based on ROESY and COSY experiments (see Figure S4 and S5 below).

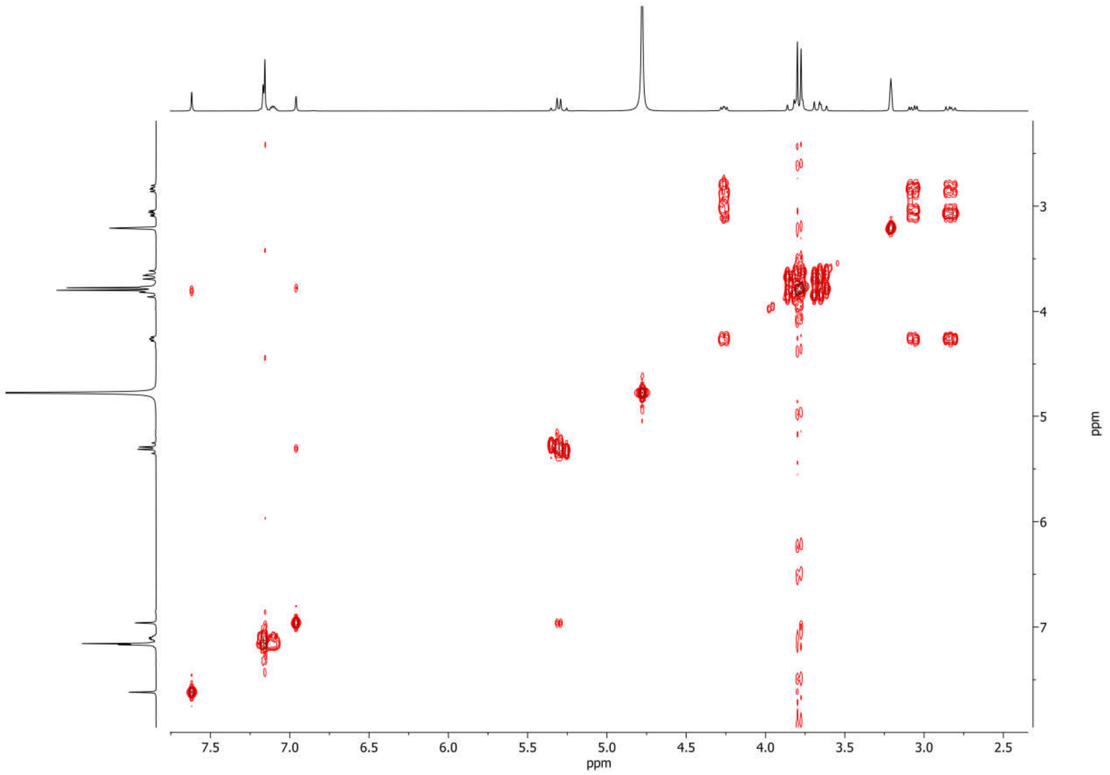

Figure S4. COSY (400 MHz) spectrum of **NVoc-FGG** in CD_3OD.

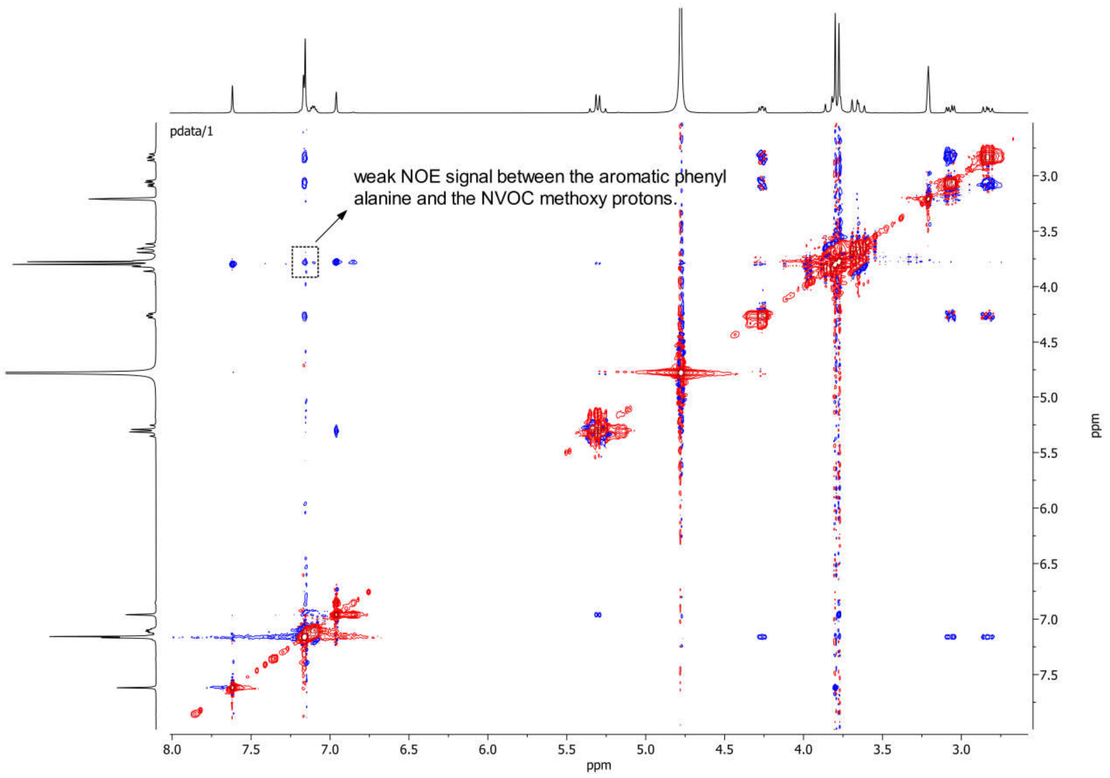

Figure S5. ROESY (400 MHz) spectrum of **NVoc-FGG** in CD_3OD.

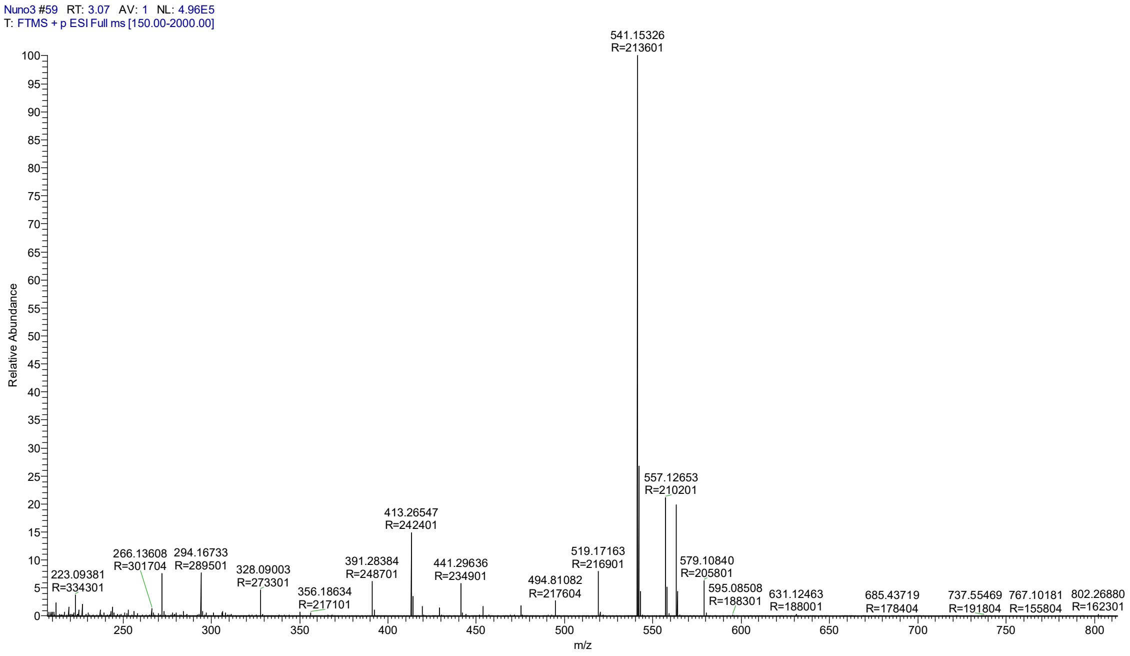

Figure S6. HRMS (ESI) spectrum of **NVoc-FGG**.

2. Additional experiments

2.1. Direct UV/vis absorption titration

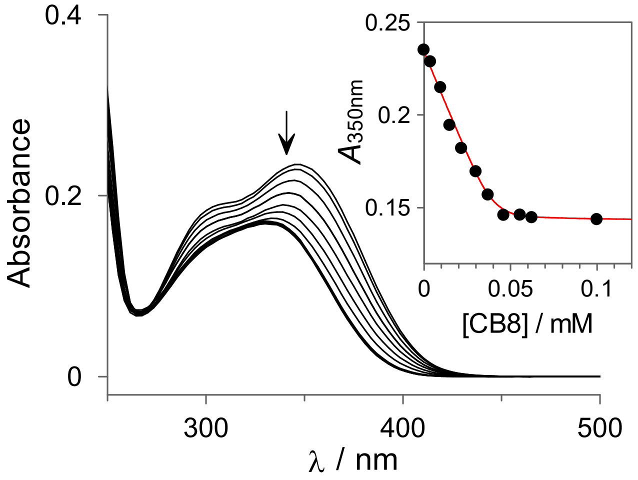

Figure S7. UV/vis absorption spectra of **NVoc-FGG** (40 μM) upon addition of increasing amounts of CB8 in water at neutral pH. The inset shows the absorbance variations at 350 nm represented against the CB8 concentration and the respective fitting to a 1:1 binding model (red line). The fitting returned a binding constant of 2.7×10^{6} M^{-1}.

2.2. Competitive displacement titration

A competitive titration experiment (Figure S8), using the 3-amino-1-adamantanol **S1** (*K* = 1.4× 10^7 M^{-1})[1] as competitor, was employed to confirm the binding constant of **NVoc-FGG** with CB8. The mathematical formalism employed to fit the data from spectroscopic competitive titrations (i.e., indicator displacement assays) has been previously described elsewhere.[1]

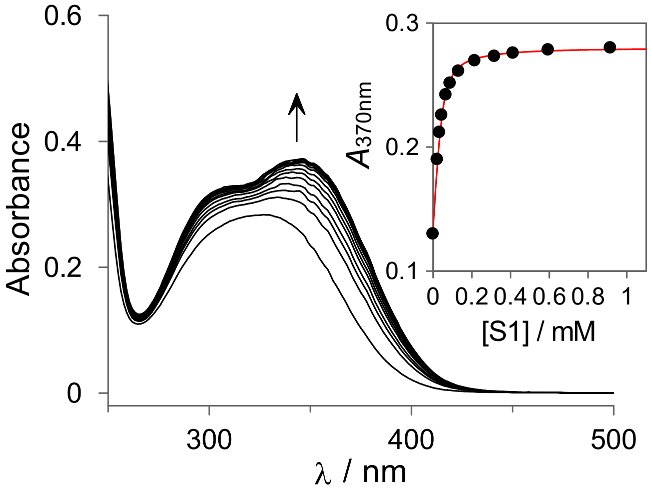

Figure S8. UV/vis absorption spectra of **NVoc-FGG** (55 μM) in the presence of 52 μM of CB8 upon addition of increasing concentrations of **S1**. All solutions were prepared in water and the spectra were acquired at 23°C. Data fitting to a competitive binding model returned an association constant of 2.9×10^6 M^{-1} for the **NVoc-FGG**@CB8 complex, which agrees very well with that obtained by direct titration (Figure S7) and ITC (see main text).

2.3. Isothermal titration calorimetry of NVoc-FGG/CB8 in buffered solution

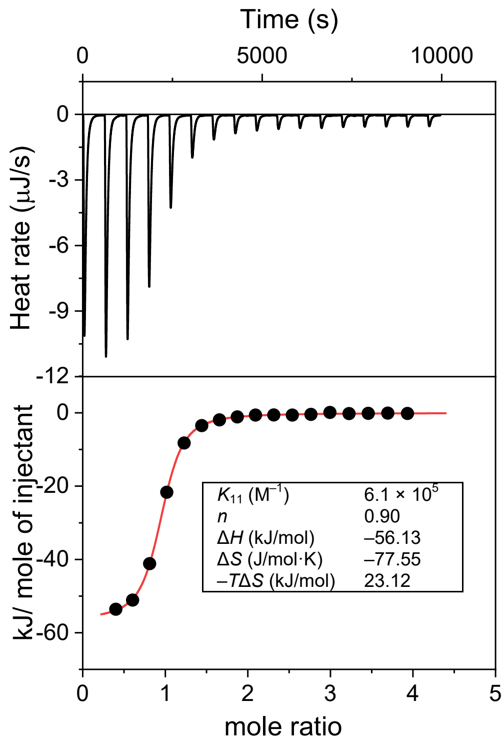

Figure 9. ITC curve for the titration of CB8 (80 μM) with aliquots of a stock solution of **NVoc-FGG** (1.5 mM) at 25°C. The experiment was performed in 10 mM phosphate-buffered water solution (pH 7.4).

2.4. Mass spectra of the inclusion complexes

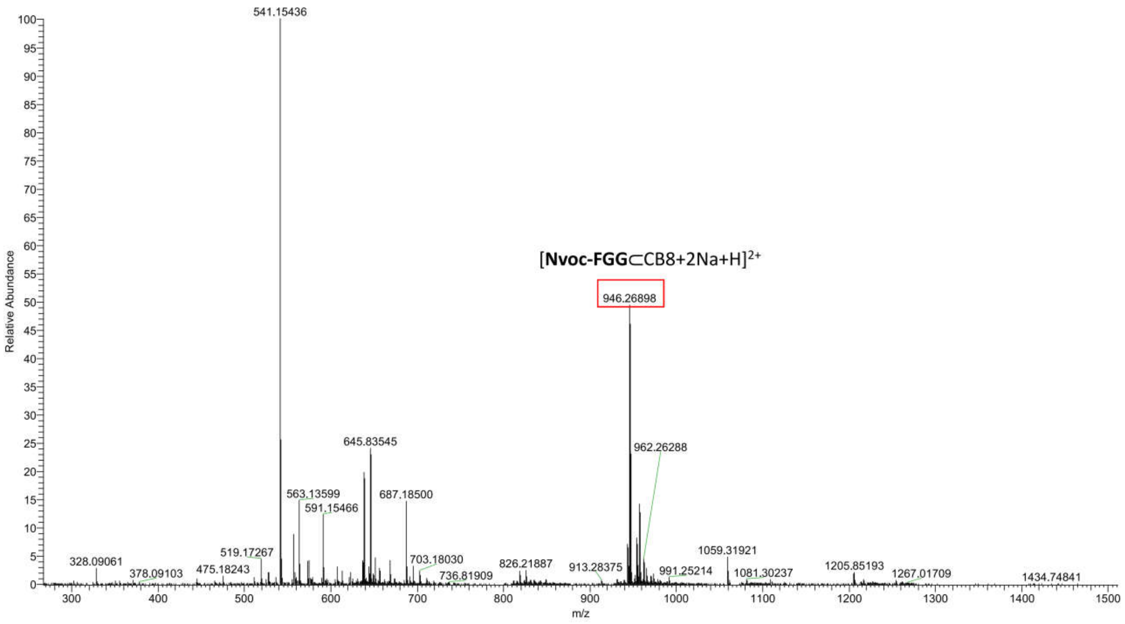

Figure S10. Mass spectrum of **NVoc-FGG**@CB8 in water.

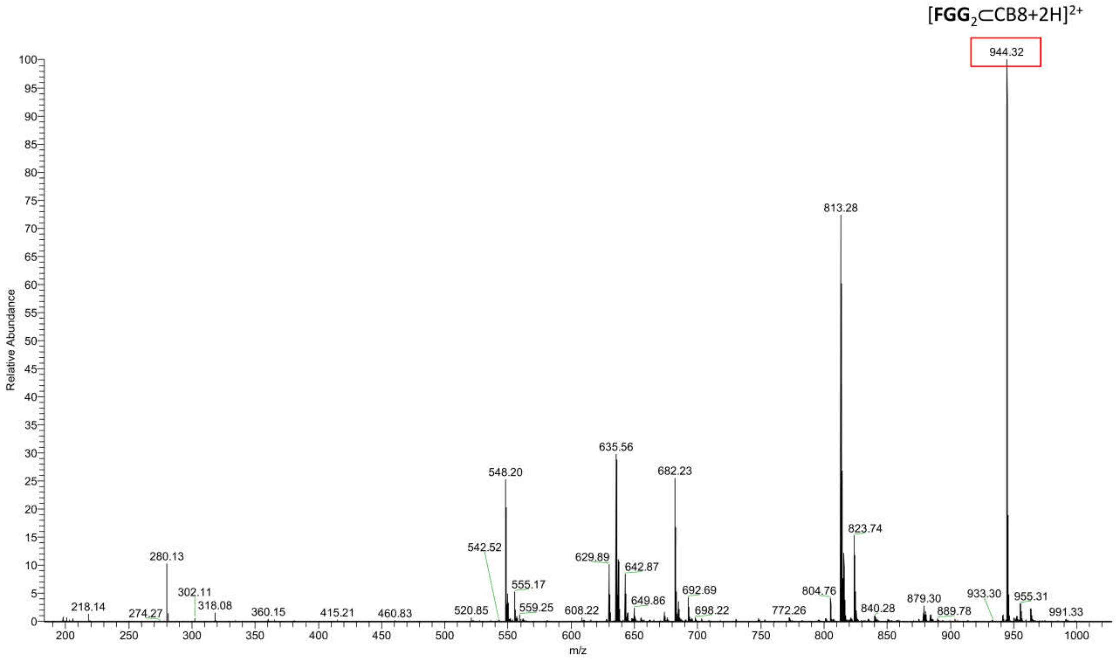

Figure S11. Mass spectrum of $\mathbf{FGG_2}$@CB8 in water.

2.5. Phenylalanine (Phe) / NVoc-Phe interactions with CB8

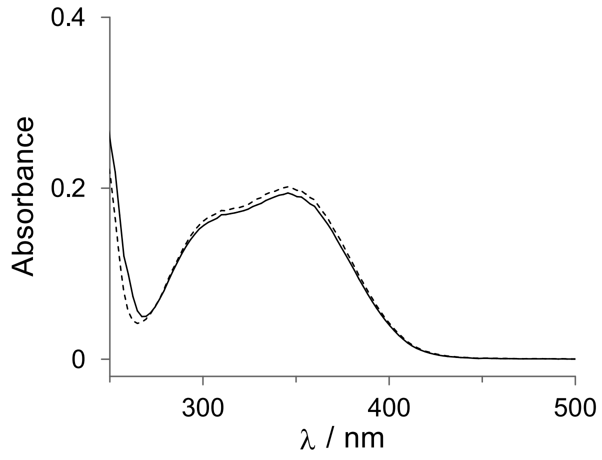

Figure S12. UV/vis absorption spectra of NVoc-phenylalanine (**NVoc-Phe**; 40 μM) in the absence (dashed line) and in the presence of 100 μM of CB8 (full line).

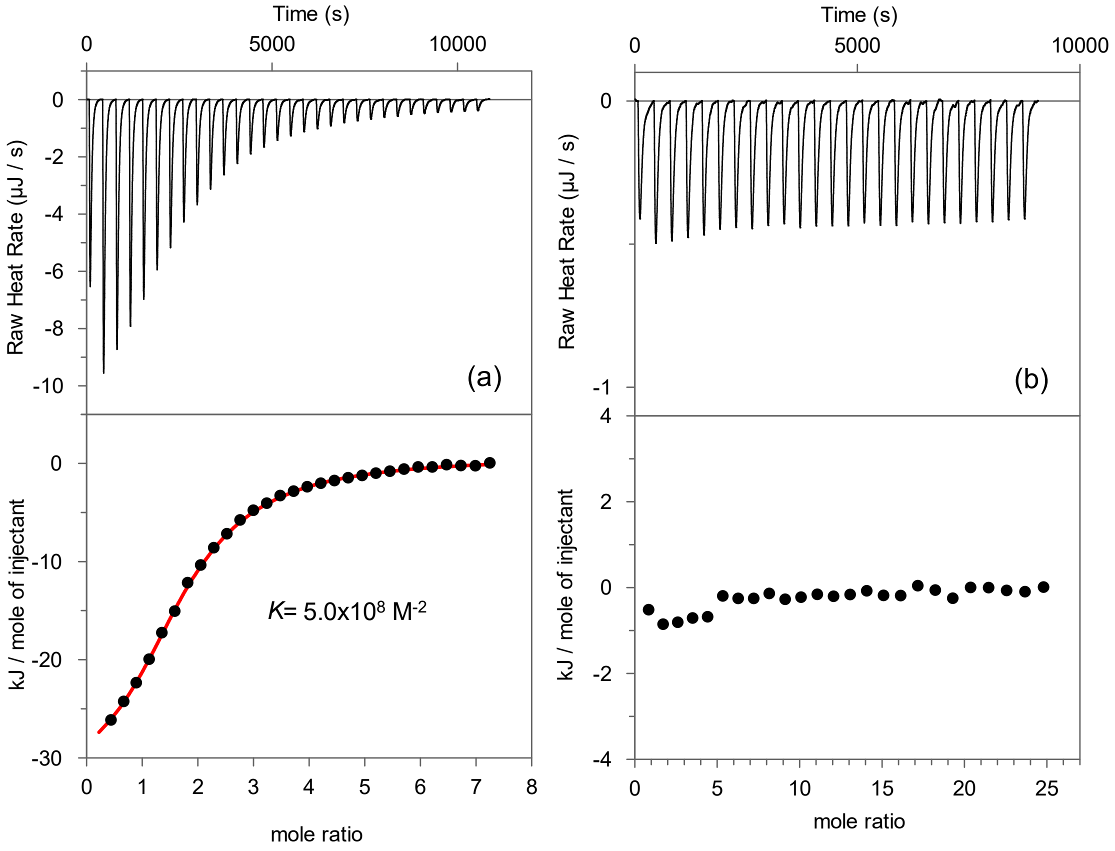

Figure S13. Isothermal titration calorimetry (ITC) data for (a) the titration of 100 μM CB8 with 4.8 mM of phenylalanine (**Phe**) in water and (b) for 10 μM CB8 with 820 μM of **NVoc-Phe** in neutral water.

2.6. NMR characterization of the inclusion complexes

The assignment of the ^{1}H NMR signals corresponding to the **NVoc-FGG**@CB8 inclusion complex revealed to be challenging due to the existence of **NVoc-FGG** rotamers in slow exchange on the chemical shift time scale (Scheme S1). The existence of slow exchange rotamers in carbamate derivatives was already documented.[2] For unbound **NVoc-FGG** the splitting of the signals corresponding to protons *a*, *b*, and *g* (see Scheme S1 and Figure S14) provides a first hint on this behaviour. However, it is worth noting that the rotamers are not equally populated, which, together with the small chemical shift differences between the two conformational isomers, precluded further structural elucidation.

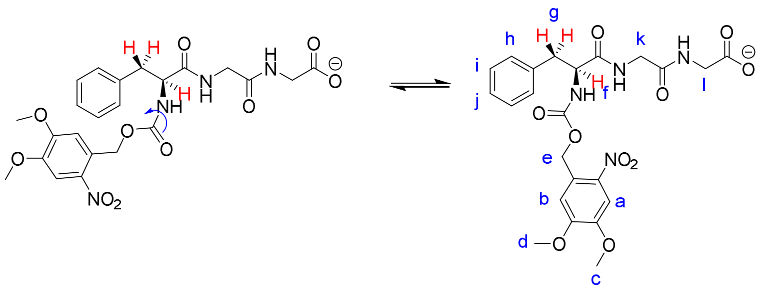

Scheme S1. Equilibrium between two **NVoc-FGG** rotamers.

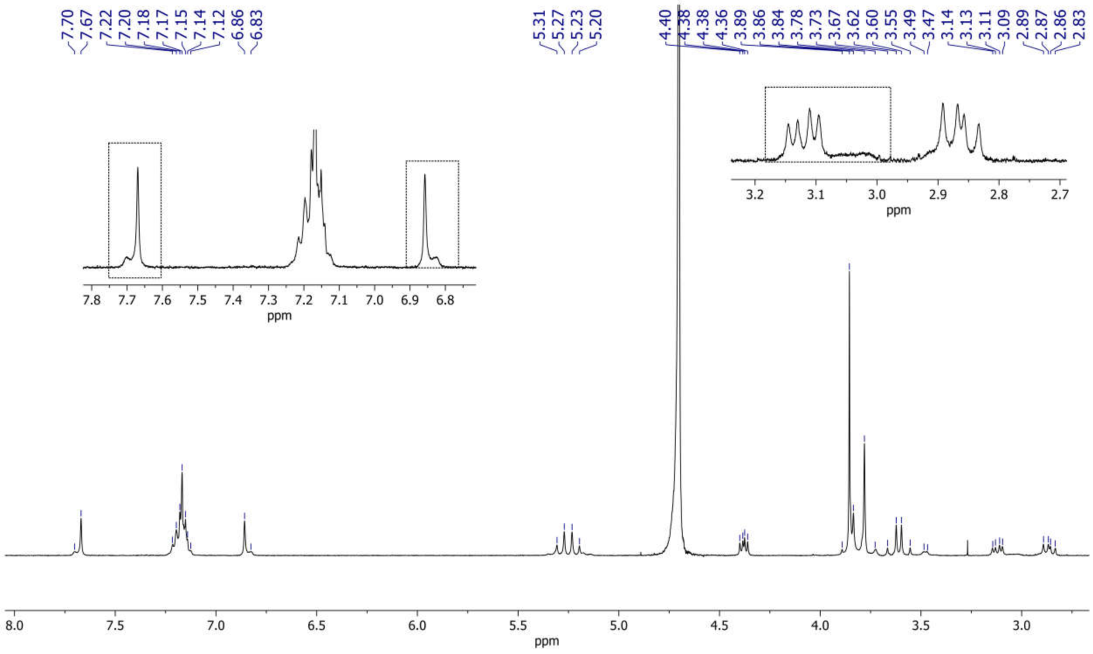

Figure S14. ^{1}H NMR (400 MHz) spectra of **NVoc-FGG** (500 μM) in D_2O.

The observation of the *rotameric* equilibrium became even more evident upon complexation of **NVoc-FGG** with CB8. As can be seen in Figure S15, two sets of signals are clearly observed for the protons *a*, *b*, and *g* (it is worth noting that the *g* protons are not magnetically equivalent and thus 4 signals are observed). Integration of these signals leads to a relative distribution of *ca*. 55:45 for the two rotamers.

The ROESY spectrum obtained for the **NVoc-FGG**@CB8 inclusion complex is shown in Figure S16. A second ROESY spectrum acquired for a sample containing ca. 0.5 equiv. of CB8 (Figure S17) allows to take advantage of the slow exchange at the NMR time scale to complete the assignment the ^{1}H NMR signals.

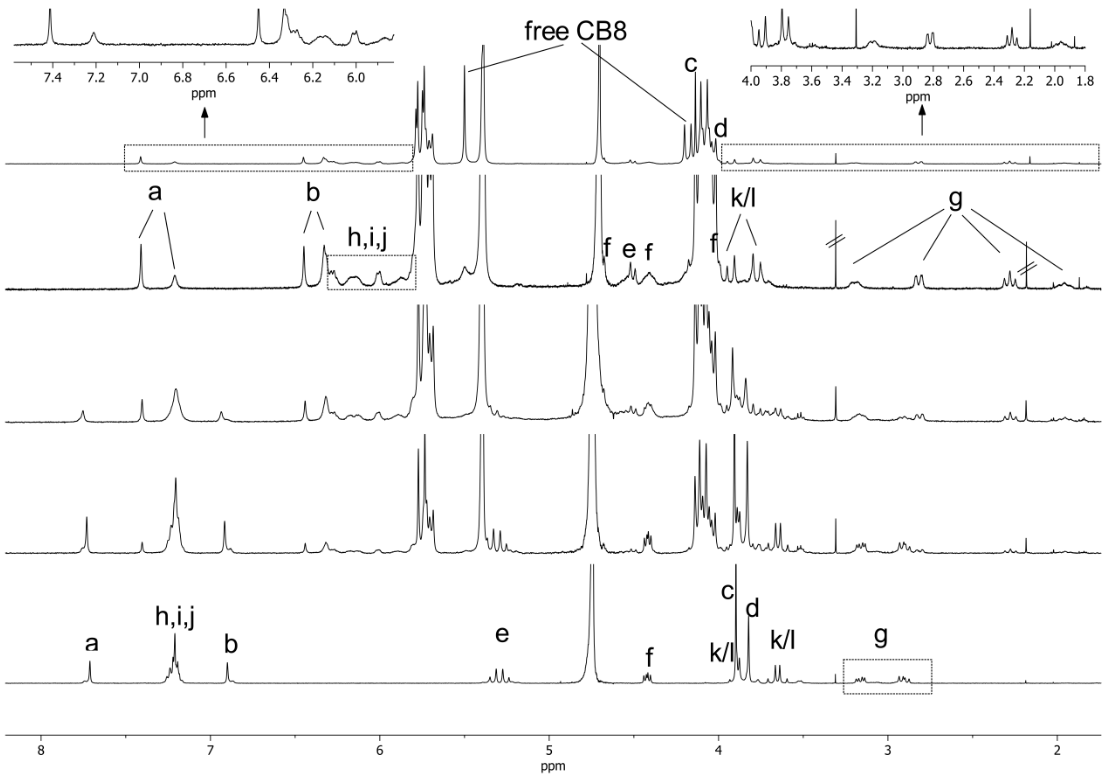

Figure S15. ^{1}H NMR (400 MHz) spectra of **NVoc-FGG** (200 μM) in D_2O in the presence of increasing concentration of CB8. // denotes traces of solvent impurities. From the bottom to the top: 0, 0.25, 0.5, 1.0 and 1.2 equiv. of CB8.

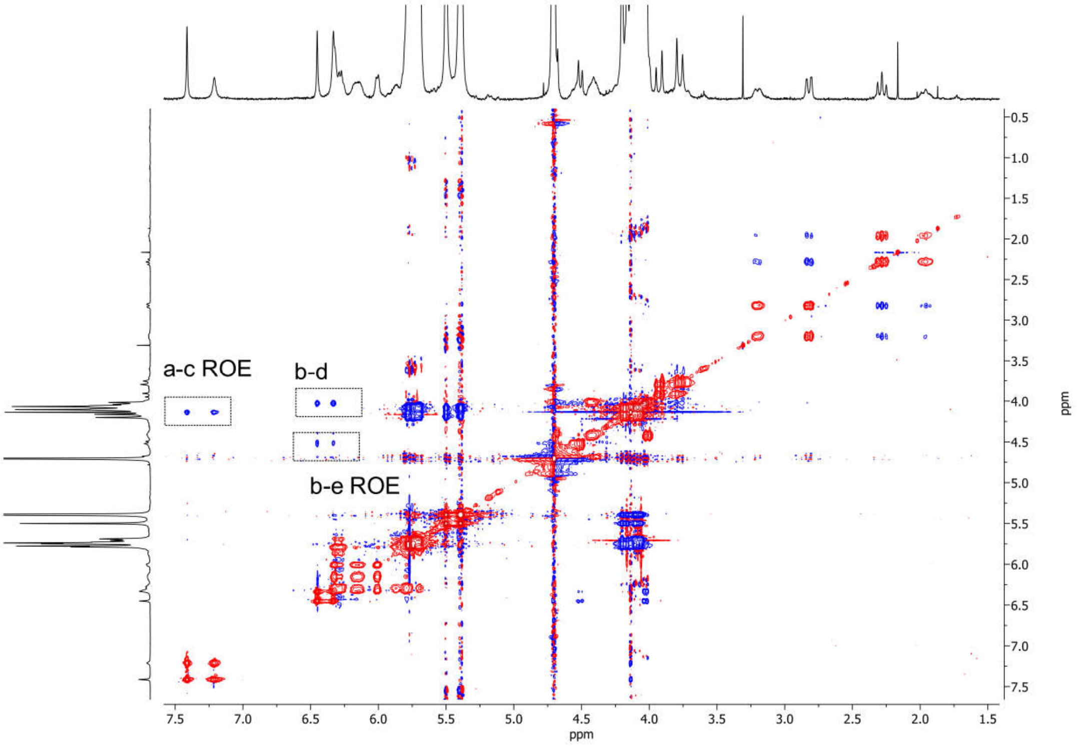

Figure S16. ROESY (400 MHz) spectrum acquired for the **NVoc-FGG**@CB8 complex (200 µM **NVoc-FGG**, 240 µM CB8) in D_2O. The mixing time was set to 300 ms.

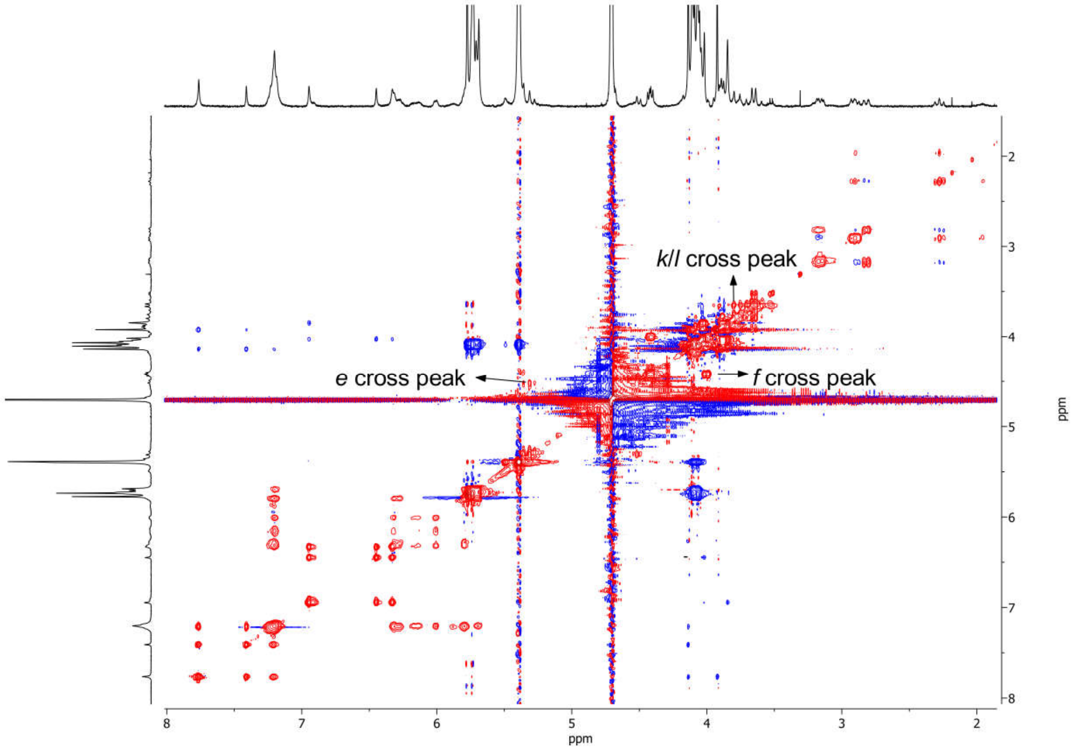

Figure S17. ROESY (400 MHz) spectrum acquired for **NVoc-FGG** (400 µM) with 0.5 equiv. CB8 (200 µM) in D_2O. The mixing time was set to 300 ms. Selected positive cross peaks are highlighted for clarity.

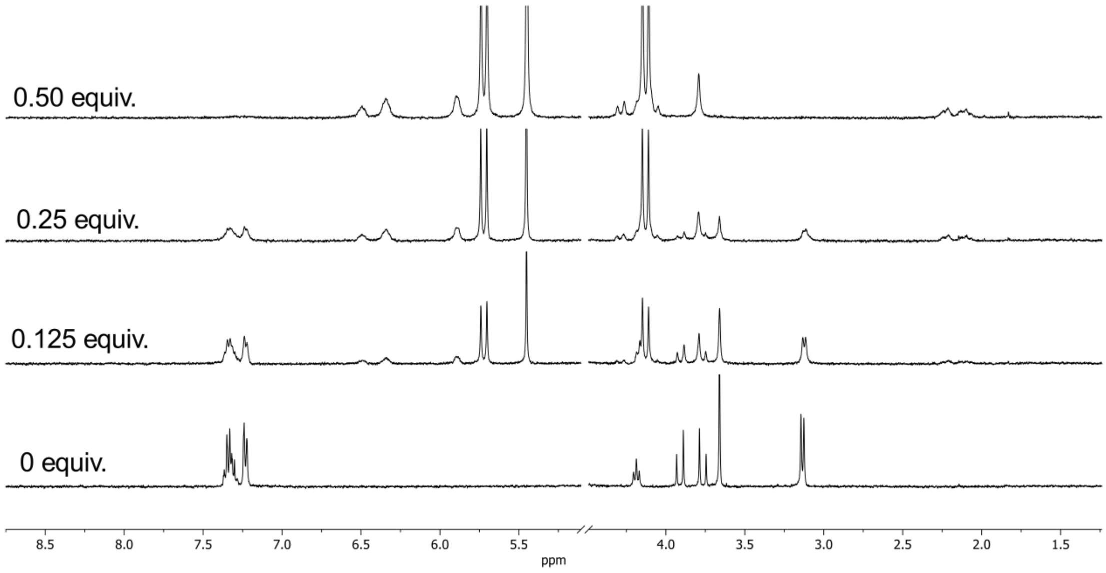

Figure S18. ^{1}H NMR (400 MHz) spectra of **FGG** (500 μM) in D_2O in the presence of increasing concentrations of CB8.

2.7. DOSY experiments

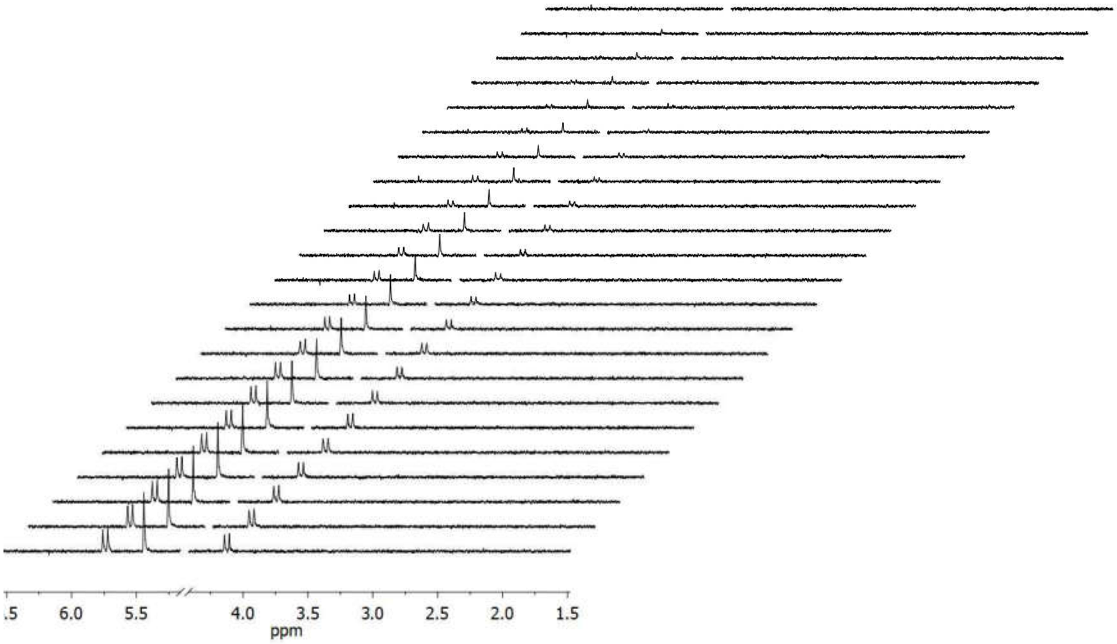

$$\frac{I}{I_0} = e^{-(\gamma\xi G\delta)^2\left(\Delta-\frac{\delta}{3}\right)D} = e^{-qD}$$

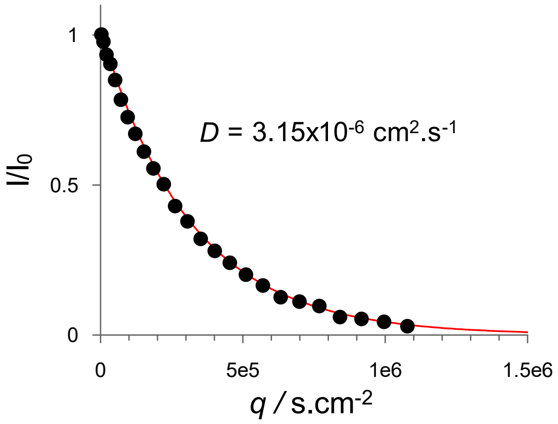

Figure S19. ^{1}H DOSY-NMR spectra (400 MHz) of 100 µM CB8 in D_2O. The normalized integrals show a mono-exponential decay when represented against parameter *q* (see above the Stejskal-Tanner equation).

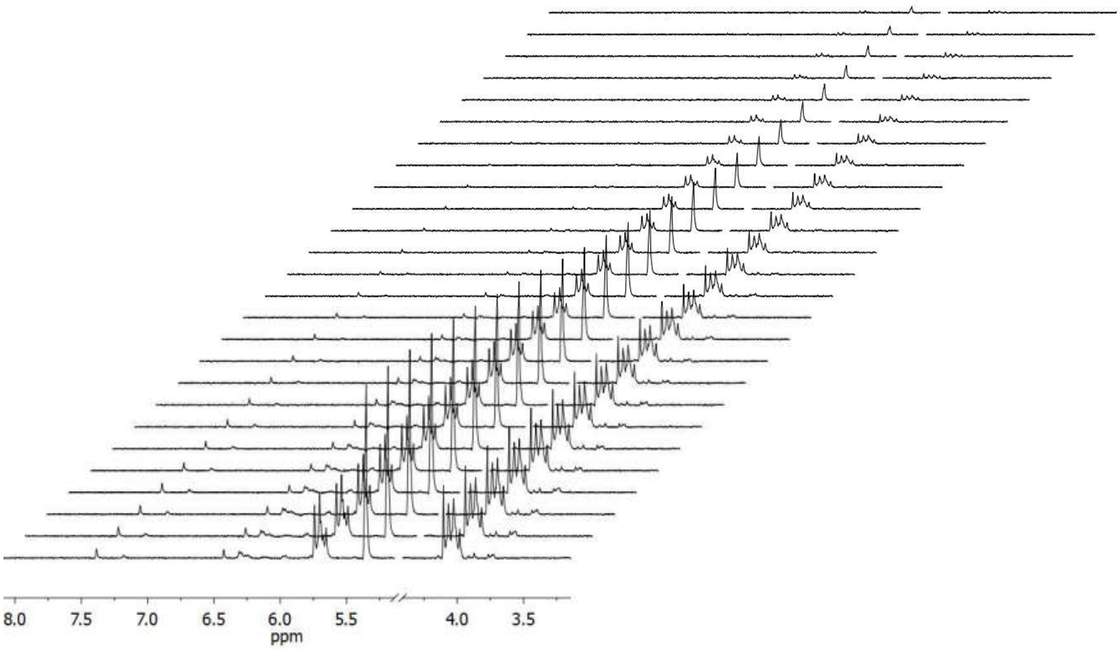

$$\frac{I}{I_0} = e^{-(\gamma \xi G \delta)^2\left(\Delta - \frac{\delta}{3}\right)D} = e^{-qD}$$

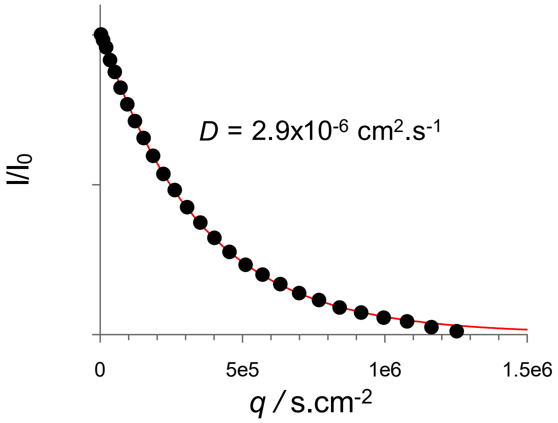

Figure S20. ^{1}H DOSY-NMR spectra (400 MHz) of 100 µM of CB8 with 200 µM of **NVoc-FGG** in D_2O. The normalized integrals (from CB8 protons) show a mono-exponential decay when represented against parameter *q* (see above the Stejskal-Tanner equation).

2.8. Photochemistry

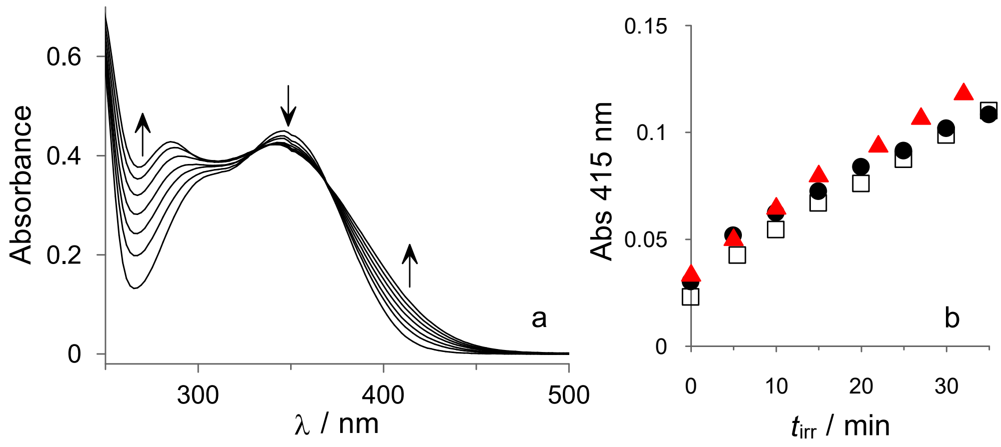

Figure S21. (**a**) Spectral variations on irradiation at 366 nm with a 200 W Xe-Hg lamp (I_0 = 5.8 × 10^{-8} Einstein s^{-1}) of **NVoc-FGG** (72μM) in 10 mM phosphate buffer (pH = 7.0) at 23°C. The same experiment was repeated in pure water at 23°C and in 10 mM of phosphate buffer (pH 7.0) at 37°C (**b**) Plot of the absorbance, at 415 nm, against the irradiation time for the different experimental conditions: (closed black circles) - 10 mM phosphate buffer (pH 7.0) at 23°C; (open squares) - pure water at 23°C; (closed red triangles) - 10 mM phosphate buffer (pH 7.0) at 37°C.

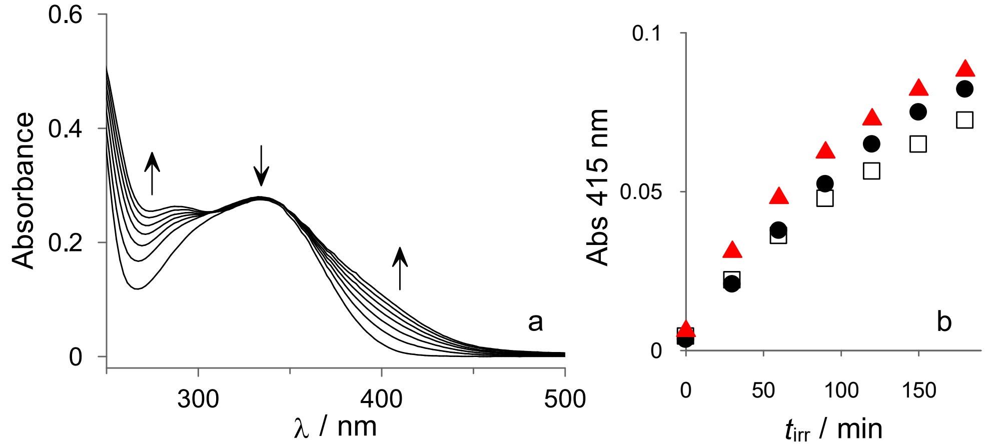

Figure S22. (**a**) Spectral variations observed upon irradiation at 366 nm with a 200 W Xe-Hg lamp (I_0 = 5.8 × 10^{-8} Einstein s^{-1}) of **NVoc-FGG** (57 μM) in the presence of 80 μM of CB8 in 10 mM of phosphate buffer (pH 7.0) at 23°C. The same experiment was repeated in pure water at 23°C and in 10 mM of phosphate buffer (pH 7.0) at 37°C (**b**) Plot of the absorbance, at 415 nm, against the irradiation time for the different experimental conditions: (closed black circles) - 10 mM of phosphate buffer (pH 7.0) at 23°C; (open squares) - pure water at 23°C; (closed red triangles) - 10 mM of phosphate buffer (pH 7.0) at 37°C.

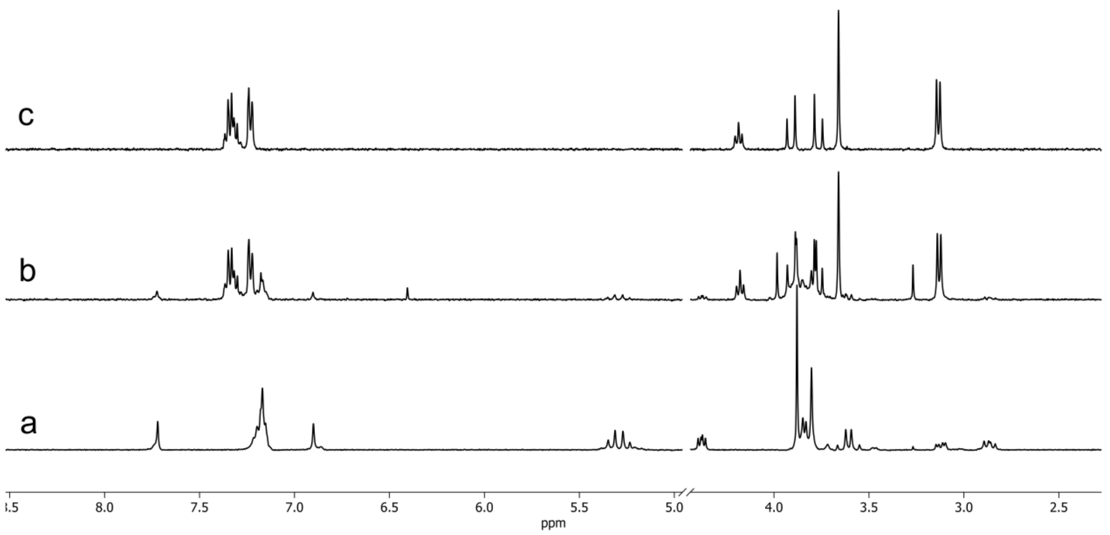

Figure S23. ^{1}H NMR (400 MHz) spectra of (a) **NVoc-FGG** (500 μM) before irradiation and (b) after photolysis with a 200 W Xe-Hg lamp (>300 nm). (c) ^{1}H NMR spectrum of **FGG** (500 μM) for comparison. This experiment shows the clean formation of **FGG** upon irradiation of **NVoc-FGG**.

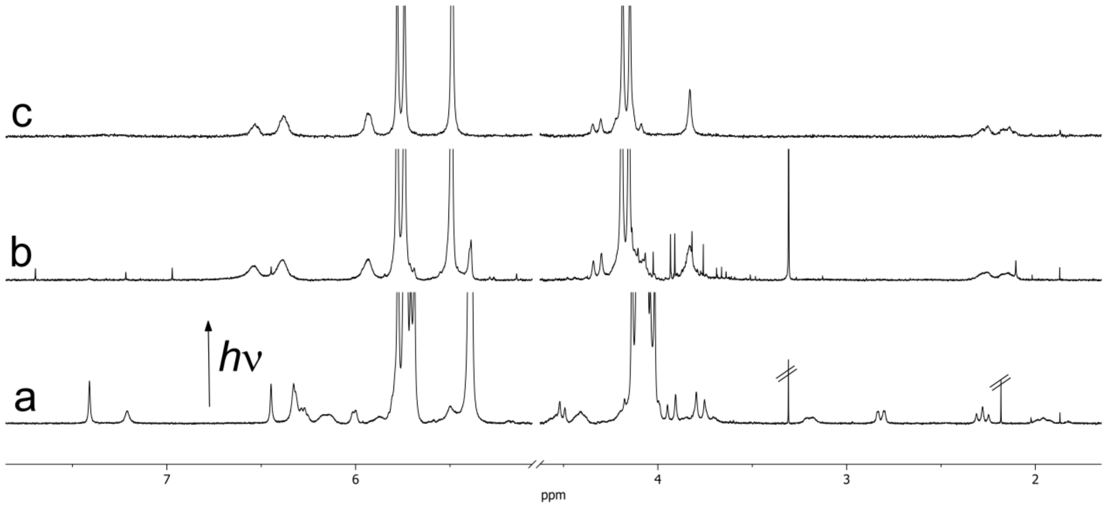

Figure S24. ^{1}H NMR (400 MHz) spectra of **NVoc-FGG** (400 μM) in the presence of 0.5 equiv of CB8 (200 μM) in D_2O (a) before and (b) after photolysis at >300 nm with a 200 W Xe-Hg lamp. Spectrum (c) corresponds to a solution of 500 μM of **FGG** with 500 μM CB8 and is used for comparison. All spectra were acquired in D_2O at 25°C.

3. References

1. Ferreira, P.; Ventura, B.; Barbieri, A.; Da Silva, J. P.; Laia, C. A. T.; Parola, A. J.; Basílio, N., A Visible–Near-Infrared Light-Responsive Host–Guest Pair with Nanomolar Affinity in Water. *Chem.–Eur. J.* **2019**, *25* (14), 3477-3482.

2. Hu, D. X.; Grice, P.; Ley, S. V., Rotamers or Diastereomers? An Overlooked NMR Solution. *J. Org. Chem.* **2012**, *77* (11), 5198-5202.
